# Supplementary material for: Update on Distribution and Conservation Status of Amphibians in the Democratic People’s Republic of Korea: Conclusions Based on Field Surveys, Environmental Modelling, Molecular Analyses and Call Properties
Source: Animals (Basel). 2021 Jul 9;11(7):2057. doi: 10.3390/ani11072057 (PMC8300379; doi:10.3390/ani11072057)
Supplement: Supplementary file 1 [file animals-11-02057-s001.zip › animals-1217986-supplementary.pdf]

animals

MDPI

Article

Update on Distribution and Conservation Status of Amphibians in the Democratic People's Republic of Korea: Conclusions Based on Field Surveys, Environmental Modelling, Molecular Analyses and Call Properties

Amaël Borzée [1,2,*], Spartak N. Litvinchuk [3,4], Kyongsim Ri [5], Desiree Andersen [6], Tu Yong Nam [7], Gwang Hyok Jon [8], Ho Song Man [9], Jong Sik Choe [9], Sera Kwon [6], Siti N. Othman [6], Kevin Messenger [10], Yoonhyuk Bae [1,6], Yucheol Shin [1], Ajoung Kim [6], Irina Maslova [11], Jennifer Luedtke [2,12], Louise Hobin [2], Nial Moores [13], Bernhard Seliger [14], Felix Glenk [14] and Yikweon Jang [15]

[1] Laboratory of Animal Behaviour and Conservation, College of Biology and the Environment, Nanjing Forestry University, 159 Longpan Rd, Nanjing 210037, China; gyyh0303@gmail.com (Y.B.); brongersmai2@gmail.com (Y.S.)
[2] Amphibian Specialist Group, IUCN Species Survival Commission, Toronto, ON L5A, Canada; jluedtke@amphibians.org (J.L.); lhobin@amphibians.org (L.H.)
[3] Institute of Cytology, Russian Academy of Sciences, Tikhoretsky pr. 4, 194064 St. Petersburg, Russia; litvinchukspartak@yandex.ru
[4] Department of Zoology and Physiology, Dagestan State University, Gadzhiyev str. 43-a, Makhachkala, 3367000 Dagestan, Russia
[5] Department of International Economic Cooperation, Ministry of Land and Environment Protection, Pyongyang, Democratic People's Republic of Korea
[6] Interdisciplinary Program of Eco Creative, Ewha Womans University, Seoul 03760, Korea; desireeka93@hotmail.com (D.A.); sera0622@hanmail.net (S.K.); dy.othman@gmail.com (S.N.O.); ajkim129@gmail.com (A.K.)
[7] Institute of Zoology, State Academy of Science, Daesong-dong, Daesong District, Pyongyang, Democratic People's Republic of Korea
[8] Department of Ecology, State Academy of Science, Daesong-dong, Daesong District, Pyongyang, Democratic People's Republic of Korea
[9] Department of Ecology, Life Science College, Kim Il Sung University, Ryongnam-dong, Daesong-dong, Daesong District, Pyongyang, Democratic People's Republic of Korea
[10] Herpetology and Applied Conservation Lab, College of Biology and the Environment, Nanjing Forestry University, 159 Longpan Rd, Nanjing 210037, China; herpsrule2@aol.com
[11] Federal Scientific Center of the East Asia Terrestrial Biodiversity, Far Eastern Branch of Russian Academy of Sciences, 690022 Vladivostok, Russia; irinarana@yandex.ru
[12] Re:wild, Austin, TX 78746, USA
[13] Birds Korea, 101-1902, Hyundai I Park, Busan 48559, Korea; nial.moores@birdskorea.org
[14] Hanns Seidel Foundation, Seoul 04419, Korea; bjseliger@yahoo.de (B.S.); glenk@hss.de (F.G.)
[15] Department of Life Sciences and Division of EcoScience, Ewha Womans University, Seoul 03760, Korea; jangy@ewha.ac.kr
* Correspondence: amaelborzee@gmail.com

Citation: Borzée, A.; Litvinchuk, S.N.; Ri, K.; Andersen, D.; Nam, T.Y.; Jon, G.H.; Man, H.S.; Choe, J.S.; Kwon, S.; Othman, S.N.; et al. Update on Distribution and Conservation Status of Amphibians in the Democratic People's Republic of Korea: Conclusions Based on Field Surveys, Environmental Modelling, Molecular Analyses and Call Properties. *Animals* **2021**, *11*, 2057. https://doi.org/10.3390/ani11072057

Academic Editor: Jesse Grismer and Andreas Maletzky

Received: 26 April 2021
Accepted: 1 July 2021
Published: 9 July 2021

Publisher's Note: MDPI stays neutral with regard to jurisdictional claims in published maps and institutional affiliations.

Copyright: © 2021 by the authors. Licensee MDPI, Basel, Switzerland. This article is an open access article distributed under the terms and conditions of the Creative Commons Attribution (CC BY) license (http://creativecommons.org/licenses/by/4.0/).

1.Supplementary files

1.1. Supplementary tables

1.1.1. Supplementary Table S1

Dataset containing GPS coordinates. Dataset for all associated with species for all the datapoints used in the models in this study. This table contains 24193 datapoints.

1.1.2. Supplementary Table S2

GPS coordinates for all geolocalised amphibian presence in DPR Korea. Dataset provided in this study and extracted from the literature. Please see methods for species specific references.

Species	Latitude	Longitude	Species	Latitude	Longitude
Dryophytes japonicus	42.5009	130.501999	*Onychodactylus*	39.147499	127.445999
Dryophytes japonicus	42.5667	130.509995	*Onychodactylus*	39.147499	127.445999
Pelophylax nigromaculatus	42.5009	130.501999	*Onychodactylus*	39.147499	127.445999
Bombina orientalis	42.3573	130.557999	*Onychodactylus*	39.147499	127.445999
Bombina orientalis	42.419399	129.699997	*Onychodactylus*	39.147499	127.445999
Bombina orientalis	42.356499	130.557007	*Onychodactylus*	39.147499	127.445999
Dryophytes japonicus	42.3582	130.561005	*Onychodactylus*	39.147499	127.445999
Dryophytes japonicus	42.345699	130.582993	*Onychodactylus*	39.147499	127.445999
Dryophytes japonicus	42.415298	129.699005	*Onychodactylus*	39.147499	127.445999
Dryophytes japonicus	42.345001	130.580002	*Onychodactylus*	39.147499	127.445999
Dryophytes japonicus	42.361328	129.669572	*Onychodactylus*	39.147499	127.445999
Pelophylax nigromaculatus	42.358299	130.561005	*Onychodactylus*	39.147499	127.445999
Pelophylax nigromaculatus	42.345699	130.582993	*Onychodactylus*	39.147499	127.445999
Pelophylax nigromaculatus	42.4184	129.701004	*Onychodactylus*	39.147499	127.445999
Pelophylax nigromaculatus	42.370098	130.432999	*Onychodactylus*	39.147499	127.445999
Pelophylax nigromaculatus	42.3825	130.455002	*Onychodactylus*	39.147499	127.445999
Pelophylax nigromaculatus	42.379002	130.412003	*Onychodactylus*	39.147499	127.445999
Pelophylax nigromaculatus	42.390301	130.522003	*Onychodactylus*	39.147499	127.445999
Pelophylax nigromaculatus	42.364498	130.356003	*Onychodactylus*	39.147499	127.445999
Pelophylax nigromaculatus	42.367901	130.522003	*Onychodactylus*	39.147499	127.445999
Pelophylax nigromaculatus	42.346401	130.550995	*Onychodactylus*	39.147499	127.445999
Pelophylax nigromaculatus	42.348801	130.207993	*Onychodactylus*	39.147499	127.445999
Pelophylax nigromaculatus	42.339199	130.242996	*Onychodactylus*	39.147499	127.445999
Pelophylax nigromaculatus	42.352001	130.486999	*Onychodactylus*	39.147499	127.445999
Pelophylax nigromaculatus	42.384499	130.240997	*Onychodactylus*	39.147499	127.445999
Pelophylax nigromaculatus	42.338799	130.391007	*Onychodactylus*	39.147499	127.445999
Pelophylax nigromaculatus	42.393002	130.498001	*Onychodactylus*	39.147499	127.445999
Pelophylax nigromaculatus	42.382198	130.257004	*Onychodactylus*	39.147499	127.445999
Pelophylax nigromaculatus	42.350601	130.358002	*Onychodactylus*	39.147499	127.445999
Pelophylax nigromaculatus	42.441898	129.744003	*Onychodactylus*	39.147499	127.445999
Pelophylax nigromaculatus	42.441898	129.744003	*Onychodactylus*	39.147499	127.445999
Pelophylax nigromaculatus	42.441898	129.744003	*Onychodactylus*	39.147499	127.445999
Pelophylax nigromaculatus	42.441898	129.744003	*Onychodactylus*	39.147499	127.445999
Pelophylax nigromaculatus	42.441898	129.744003	*Onychodactylus*	39.147499	127.445999
Pelophylax nigromaculatus	42.441898	129.744003	*Onychodactylus*	39.147499	127.445999

Pelophylax nigromaculatus	42.441898	129.744003	*Onychodactylus*	39.147499	127.445999
Pelophylax nigromaculatus	42.441898	129.744003	*Onychodactylus*	39.147499	127.445999
Pelophylax nigromaculatus	42.441898	129.744003	*Onychodactylus*	39.147499	127.445999
Pelophylax nigromaculatus	42.441898	129.744003	*Onychodactylus*	39.147499	127.445999
Rana dybowskii	42.419899	129.699997	*Onychodactylus*	39.147499	127.445999
Rana dybowskii	42.345001	130.371994	*Onychodactylus*	39.147499	127.445999
Rana uenoi-dybowskii	42.419899	129.699997	*Onychodactylus*	39.147499	127.445999
Rana uenoi-dybowskii	42.345001	130.371994	*Onychodactylus*	39.147499	127.445999
Bombina orientalis	42.3251	130.453003	*Onychodactylus*	39.147499	127.445999
Bombina orientalis	42.2812	129.225006	*Onychodactylus*	39.147499	127.445999
Bombina orientalis	42.222599	129.214996	*Onychodactylus*	39.147499	127.445999
Bombina orientalis	42.286598	130.304993	*Onychodactylus*	39.147499	127.445999
Bombina orientalis	42.325298	130.451004	*Onychodactylus*	39.147499	127.445999
Bombina orientalis	42.3256	130.451004	*Onychodactylus*	39.147499	127.445999
Bombina orientalis	42.240501	130.358993	*Onychodactylus*	39.147499	127.445999
Bombina orientalis	42.222599	129.214996	*Onychodactylus*	39.147499	127.445999
Bufo gargarizans	42.273701	130.294998	*Onychodactylus*	39.147499	127.445999
Bufo gargarizans	42.2742	130.296005	*Onychodactylus*	39.147499	127.445999
Dryophytes japonicus	42.304199	130.389999	*Onychodactylus*	39.147499	127.445999
Dryophytes japonicus	42.2659	129.223007	*Onychodactylus*	39.147499	127.445999
Pelophylax nigromaculatus	42.304001	130.389999	*Onychodactylus*	39.147499	127.445999
Pelophylax nigromaculatus	42.2328	129.225006	*Onychodactylus*	39.147499	127.445999
Pelophylax nigromaculatus	42.331799	130.576996	*Onychodactylus*	39.147499	127.445999
Pelophylax nigromaculatus	42.287102	130.242996	*Onychodactylus*	39.147499	127.445999
Pelophylax nigromaculatus	42.325901	130.584	*Onychodactylus*	39.147499	127.445999
Pelophylax nigromaculatus	42.312302	130.227997	*Onychodactylus*	39.147499	127.445999
Pelophylax nigromaculatus	42.292	130.207993	*Onychodactylus*	39.147499	127.445999
Pelophylax nigromaculatus	42.237202	130.248993	*Onychodactylus*	39.147499	127.445999
Pelophylax nigromaculatus	42.253502	130.225006	*Onychodactylus*	39.147499	127.445999
Pelophylax nigromaculatus	42.330101	130.218002	*Onychodactylus*	39.147499	127.445999
Pelophylax nigromaculatus	42.244999	130.281006	*Onychodactylus*	39.147499	127.445999
Pelophylax nigromaculatus	42.330799	130.375	*Onychodactylus*	39.147499	127.445999
Pelophylax nigromaculatus	42.289799	130.257004	*Onychodactylus*	39.147499	127.445999
Rana dybowskii	42.286499	130.307007	*Onychodactylus*	39.147499	127.445999
Rana dybowskii	42.2757	129.227005	*Onychodactylus*	39.147499	127.445999
Rana dybowskii	42.277199	130.298004	*Onychodactylus*	39.147499	127.445999
Rana dybowskii	42.282299	130.315002	*Onychodactylus*	39.147499	127.445999
Rana dybowskii	42.280102	130.300995	*Onychodactylus*	39.147499	127.445999
Rana uenoi-dybowskii	42.286499	130.307007	*Onychodactylus*	39.147499	127.445999
Rana uenoi-dybowskii	42.2757	129.227005	*Onychodactylus*	39.147499	127.445999
Rana uenoi-dybowskii	42.277199	130.298004	*Onychodactylus*	39.147499	127.445999
Rana uenoi-dybowskii	42.282299	130.315002	*Onychodactylus*	39.147499	127.445999

Rana uenoi-dybowskii	42.280102	130.300995	*Onychodactylus*	39.147499	127.445999
Rana dybowskii	42.130299	129.733002	*Onychodactylus*	39.147499	127.445999
Rana dybowskii	42.1628	129.188995	*Onychodactylus*	39.147499	127.445999
Rana uenoi-dybowskii	42.130299	129.733002	*Onychodactylus*	39.147499	127.445999
Rana uenoi-dybowskii	42.1628	129.188995	*Onychodactylus*	39.147499	127.445999
Bombina orientalis	41.952	128.153	*Onychodactylus*	39.147499	127.445999
Glandirana rugosa	42.0606	129.712006	*Onychodactylus*	39.147499	127.445999
Glandirana rugosa	42.0606	129.712006	*Onychodactylus*	39.147499	127.445999
Glandirana rugosa	42.0606	129.712006	*Onychodactylus*	39.147499	127.445999
Glandirana rugosa	42.0606	129.712006	*Onychodactylus*	39.147499	127.445999
Glandirana rugosa	42.0606	129.712006	*Onychodactylus*	39.147499	127.445999
Glandirana rugosa	42.0606	129.712006	*Onychodactylus*	39.147499	127.445999
Glandirana rugosa	42.0606	129.712006	*Onychodactylus*	39.147499	127.445999
Glandirana rugosa	42.0606	129.712006	*Onychodactylus*	39.147499	127.445999
Glandirana rugosa	42.0606	129.712006	*Onychodactylus*	39.147499	127.445999
Glandirana rugosa	42.0606	129.712006	*Onychodactylus*	39.147499	127.445999
Glandirana rugosa	42.0606	129.712006	*Onychodactylus*	39.147499	127.445999
Glandirana rugosa	42.0606	129.712006	*Onychodactylus*	39.147499	127.445999
Rana dybowskii	41.962	128.16	*Onychodactylus*	39.147499	127.445999
Rana dybowskii	41.969601	129.063995	*Onychodactylus*	39.147499	127.445999
Rana uenoi-dybowskii	41.969601	129.063995	*Onychodactylus*	39.147499	127.445999
Dryophytes japonicus	41.925408	129.424442	*Onychodactylus*	39.147499	127.445999
Onychodactylus	41.874802	128.212997	*Onychodactylus*	39.147499	127.445999
Onychodactylus	41.877399	128.938995	*Onychodactylus*	39.147499	127.445999
Pelophylax nigromaculatus	41.925701	128.153	*Onychodactylus*	39.147499	127.445999
Rana dybowskii	41.8666	128.261002	*Onychodactylus*	39.147499	127.445999
Rana uenoi-dybowskii	41.8666	128.261002	*Onychodactylus*	39.147499	127.445999
Bombina orientalis	41.785301	129.768997	*Onychodactylus*	39.147499	127.445999
Bombina orientalis	41.785301	129.768997	*Onychodactylus*	39.147499	127.445999
Bombina orientalis	41.785301	129.768997	*Onychodactylus*	39.147499	127.445999
Bombina orientalis	41.785301	129.768997	*Onychodactylus*	39.147499	127.445999
Bombina orientalis	41.785301	129.768997	*Onychodactylus*	39.147499	127.445999
Bombina orientalis	41.785301	129.768997	*Onychodactylus*	39.147499	127.445999
Bombina orientalis	41.785301	129.768997	*Onychodactylus*	39.147499	127.445999
Bombina orientalis	41.785301	129.768997	*Onychodactylus*	39.147499	127.445999
Dryophytes japonicus	41.800098	129.776001	*Onychodactylus*	39.147499	127.445999
Glandirana rugosa	41.785301	129.768997	*Onychodactylus*	39.147499	127.445999
Glandirana rugosa	41.785301	129.768997	*Onychodactylus*	39.147499	127.445999
Glandirana rugosa	41.785301	129.768997	*Onychodactylus*	39.147499	127.445999
Glandirana rugosa	41.785301	129.768997	*Onychodactylus*	39.147499	127.445999
Glandirana rugosa	41.785301	129.768997	*Onychodactylus*	39.147499	127.445999
Glandirana rugosa	41.785301	129.768997	*Onychodactylus*	39.147499	127.445999

Glandirana rugosa	41.785301	129.768997	*Onychodactylus*	39.147499	127.445999
Glandirana rugosa	41.785301	129.768997	*Onychodactylus*	39.147499	127.445999
Onychodactylus	41.749401	128.350006	*Onychodactylus*	39.147499	127.445999
Pelophylax nigromaculatus	41.786301	129.791	*Onychodactylus*	39.147499	127.445999
Pelophylax nigromaculatus	41.785301	129.768997	*Onychodactylus*	39.147499	127.445999
Pelophylax nigromaculatus	41.785301	129.768997	*Onychodactylus*	39.147499	127.445999
Pelophylax nigromaculatus	41.785301	129.768997	*Onychodactylus*	39.147499	127.445999
Pelophylax nigromaculatus	41.785301	129.768997	*Onychodactylus*	39.147499	127.445999
Pelophylax nigromaculatus	41.785301	129.768997	*Onychodactylus*	39.147499	127.445999
Pelophylax nigromaculatus	41.785301	129.768997	*Onychodactylus*	39.147499	127.445999
Pelophylax nigromaculatus	41.785301	129.768997	*Onychodactylus*	39.147499	127.445999
Pelophylax nigromaculatus	41.785301	129.768997	*Onychodactylus*	39.147499	127.445999
Pelophylax nigromaculatus	41.785301	129.768997	*Onychodactylus*	39.147499	127.445999
Pelophylax nigromaculatus	41.785301	129.768997	*Onychodactylus*	39.147499	127.445999
Pelophylax nigromaculatus	41.785301	129.768997	*Onychodactylus*	39.147499	127.445999
Pelophylax nigromaculatus	41.785301	129.768997	*Onychodactylus*	39.147499	127.445999
Pelophylax nigromaculatus	41.785301	129.768997	*Onychodactylus*	39.147499	127.445999
Pelophylax nigromaculatus	41.785301	129.768997	*Onychodactylus*	39.147499	127.445999
Pelophylax nigromaculatus	41.785301	129.768997	*Onychodactylus*	39.147499	127.445999
Pelophylax nigromaculatus	41.785301	129.768997	*Onychodactylus*	39.147499	127.445999
Pelophylax nigromaculatus	41.785301	129.768997	*Onychodactylus*	39.147499	127.445999
Pelophylax nigromaculatus	41.785301	129.768997	*Onychodactylus*	39.147499	127.445999
Pelophylax nigromaculatus	41.785301	129.768997	*Onychodactylus*	39.147499	127.445999
Pelophylax nigromaculatus	41.785301	129.768997	*Onychodactylus*	39.147499	127.445999
Pelophylax nigromaculatus	41.785301	129.768997	*Onychodactylus*	39.147499	127.445999
Pelophylax nigromaculatus	41.785301	129.768997	*Onychodactylus*	39.147499	127.445999
Pelophylax nigromaculatus	41.785301	129.768997	*Onychodactylus*	39.147499	127.445999
Pelophylax nigromaculatus	41.785301	129.768997	*Onychodactylus*	39.147499	127.445999
Pelophylax nigromaculatus	41.785301	129.768997	*Onychodactylus*	39.147499	127.445999
Rana dybowskii	41.788399	129.785004	*Onychodactylus*	39.147499	127.445999
Rana dybowskii	41.803101	128.320007	*Onychodactylus*	39.147499	127.445999
Rana uenoi-dybowskii	41.788399	129.785004	*Onychodactylus*	39.147499	127.445999
Rana uenoi-dybowskii	41.803101	128.320007	*Onychodactylus*	39.147499	127.445999
Salamandrella tridactyla	41.766899	129.106003	*Onychodactylus*	39.147499	127.445999
Dryophytes japonicus	41.548726	128.486679	*Onychodactylus*	39.147499	127.445999
Rana dybowskii	41.558201	128.384995	*Onychodactylus*	39.147499	127.445999
Rana uenoi-dybowskii	41.558201	128.384995	*Onychodactylus*	39.147499	127.445999
Salamandrella tridactyla	41.62439	128.851861	*Onychodactylus*	39.147499	127.445999
Bombina orientalis	41.408901	129.654007	*Onychodactylus*	39.147499	127.445999
Pelophylax nigromaculatus	41.4161	129.699997	*Onychodactylus*	39.147499	127.445999
Bombina orientalis	41.387402	128.173996	*Onychodactylus*	39.147499	127.445999
Bufo gargarizans	41.3862	129.630005	*Onychodactylus*	39.147499	127.445999

Dryophytes japonicus	41.376598	129.619003	*Onychodactylus*	39.147499	127.445999
Dryophytes japonicus	41.304113	127.057972	*Onychodactylus*	39.147499	127.445999
Onychodactylus	41.395699	128.541	*Onychodactylus*	39.147499	127.445999
Rana dybowskii	41.280602	128.598999	*Onychodactylus*	39.147499	127.445999
Rana dybowskii	41.387699	128.173996	*Onychodactylus*	39.147499	127.445999
Rana uenoi-dybowskii	41.280602	128.598999	*Onychodactylus*	39.147499	127.445999
Rana uenoi-dybowskii	41.387699	128.173996	*Onychodactylus*	39.147499	127.445999
Salamandrella tridactyla	41.403	128.856	*Onychodactylus*	39.147499	127.445999
Dryophytes japonicus	41.270208	128.125147	*Onychodactylus*	39.147499	127.445999
Onychodactylus	41.195999	128.472	*Onychodactylus*	39.147499	127.445999
Salamandrella tridactyla	41.241299	128.587997	*Onychodactylus*	39.147499	127.445999
Dryophytes japonicus	40.902599	129.429001	*Onychodactylus*	39.147499	127.445999
Bombina orientalis	40.837002	128.184006	*Onychodactylus*	39.147499	127.445999
Bombina orientalis	40.862	126.649002	*Onychodactylus*	39.147499	127.445999
Bombina orientalis	40.862	126.649002	*Onychodactylus*	39.147499	127.445999
Bombina orientalis	40.862	126.649002	*Onychodactylus*	39.147499	127.445999
Bombina orientalis	40.862	126.649002	*Onychodactylus*	39.147499	127.445999
Bombina orientalis	40.862	126.649002	*Onychodactylus*	39.147499	127.445999
Bombina orientalis	40.862	126.649002	*Onychodactylus*	39.147499	127.445999
Bombina orientalis	40.862	126.649002	*Onychodactylus*	39.147499	127.445999
Bombina orientalis	40.862	126.649002	*Onychodactylus*	39.147499	127.445999
Bombina orientalis	40.862	126.649002	*Onychodactylus*	39.147499	127.445999
Bombina orientalis	40.862	126.649002	*Onychodactylus*	39.147499	127.445999
Bombina orientalis	40.862	126.649002	*Onychodactylus*	39.147499	127.445999
Bombina orientalis	40.862	126.649002	*Onychodactylus*	39.147499	127.445999
Bombina orientalis	40.862	126.649002	*Onychodactylus*	39.147499	127.445999
Bombina orientalis	40.862	126.649002	*Onychodactylus*	39.147499	127.445999
Bombina orientalis	40.862	126.649002	*Onychodactylus*	39.147499	127.445999
Bombina orientalis	40.862	126.649002	*Onychodactylus*	39.147499	127.445999
Bombina orientalis	40.862	126.649002	*Onychodactylus*	39.147499	127.445999
Bombina orientalis	40.862	126.649002	*Onychodactylus*	39.147499	127.445999
Bombina orientalis	40.862	126.649002	*Onychodactylus*	39.147499	127.445999
Bufo gargarizans	40.832199	129.380005	*Onychodactylus*	39.147499	127.445999
Dryophytes japonicus	40.826099	128.179001	*Onychodactylus*	39.147499	127.445999
Glandirana emeljanovi	40.8223	128.175003	*Onychodactylus*	39.147499	127.445999
Glandirana rugosa	40.819698	129.354004	*Onychodactylus*	39.147499	127.445999
Pelophylax nigromaculatus	40.841599	127.971001	*Onychodactylus*	39.147499	127.445999
Pelophylax nigromaculatus	40.822601	128.179001	*Onychodactylus*	39.147499	127.445999
Salamandrella tridactyla	40.849701	129.416	*Onychodactylus*	39.147499	127.445999
Bufo gargarizans	40.731998	127.914001	*Onychodactylus*	39.147499	127.445999
Glandirana rugosa	40.680901	127.276001	*Onychodactylus*	39.147499	127.445999

Kaloula borealis	40.722	129.234	*Onychodactylus*	39.147499	127.445999
Dryophytes japonicus	40.454148	127.033393	*Onychodactylus*	39.147499	127.445999
Hynobius leechii	40.378399	125.119003	*Onychodactylus*	39.147499	127.445999
Onychodactylus	40.447701	127.570999	*Onychodactylus*	39.147499	127.445999
Onychodactylus	40.446999	127.341003	*Onychodactylus*	39.147499	127.445999
Rana dybowskii	40.415199	127.262001	*Onychodactylus*	39.147499	127.445999
Rana uenoi-dybowskii	40.415199	127.262001	*Onychodactylus*	39.147499	127.445999
Bombina orientalis	40.309101	125.969002	*Onychodactylus*	39.147499	127.445999
Hynobius leechii	40.231201	125.495003	*Onychodactylus*	39.147499	127.445999
Strauchbufo raddei	40.22	124.568	*Onychodactylus*	39.147499	127.445999
Strauchbufo raddei	40.2687	124.810997	*Onychodactylus*	39.147499	127.445999
Dryophytes japonicus	40.176794	127.931244	*Pelophylax chosenicus*	39.196112	125.604961
Dryophytes japonicus	40.176794	127.931244	*Pelophylax chosenicus*	39.204263	125.595751
Dryophytes japonicus	40.176794	127.931244	*Pelophylax chosenicus*	39.152199	125.762001
Dryophytes japonicus	40.176794	127.931244	*Pelophylax nigromaculatus*	39.197044	125.60599
Dryophytes japonicus	40.176794	127.931244	*Pelophylax nigromaculatus*	39.203341	125.596781
Dryophytes japonicus	40.176794	127.931244	*Pelophylax nigromaculatus*	39.215199	126.864998
Kaloula borealis	40.079601	124.899002	*Pelophylax nigromaculatus*	39.254501	125.918999
Pelophylax nigromaculatus	40.1768	127.931	*Pelophylax nigromaculatus*	39.147499	127.445999
Pelophylax nigromaculatus	40.1768	127.931	*Pelophylax nigromaculatus*	39.147499	127.445999
Pelophylax nigromaculatus	40.1768	127.931	*Pelophylax nigromaculatus*	39.147499	127.445999
Pelophylax nigromaculatus	40.1768	127.931	*Pelophylax nigromaculatus*	39.147499	127.445999
Pelophylax nigromaculatus	40.1768	127.931	*Pelophylax nigromaculatus*	39.147499	127.445999
Pelophylax nigromaculatus	40.1768	127.931	*Pelophylax nigromaculatus*	39.147499	127.445999
Pelophylax nigromaculatus	40.1768	127.931	*Pelophylax nigromaculatus*	39.147499	127.445999
Pelophylax nigromaculatus	40.1768	127.931	*Rana coreana*	39.245471	125.685587
Pelophylax nigromaculatus	40.1768	127.931	*Rana coreana*	39.195599	125.606003
Pelophylax nigromaculatus	40.1768	127.931	*Rana coreana*	39.186501	125.521004
Pelophylax nigromaculatus	40.1768	127.931	*Rana dybowskii*	39.203899	125.842003
Pelophylax nigromaculatus	40.1768	127.931	*Rana uenoi-dybowskii*	39.203899	125.842003
Pelophylax nigromaculatus	40.1768	127.931	*Rana uenoi-dybowskii*	39.1475	127.445833
Strauchbufo raddei	40.072	124.383	*Bufo gargarizans*	39.007999	125.736999
Strauchbufo raddei	40.144299	124.449997	*Bufo gargarizans*	39.078899	127.459
Strauchbufo raddei	40.131802	124.685997	*Bufo gargarizans*	39.004902	125.736999
Rana amurensis	40.1768	127.931	*Dryophytes japonicus*	39.020661	125.717334
Rana amurensis	40.1768	127.931	*Dryophytes japonicus*	39.079601	127.447998
Rana amurensis	40.1768	127.931	*Dryophytes japonicus*	39.004501	125.734001
Rana amurensis	40.1768	127.931	*Dryophytes japonicus*	39.078899	125.850998
Rana amurensis	40.1768	127.931	*Dryophytes japonicus*	39.07485	127.416658
Rana amurensis	40.1768	127.931	*Dryophytes suweonensis*	39.125534	125.726775
Rana amurensis	40.1768	127.931	*Glandirana rugosa*	39.098301	127.514
Rana amurensis	40.1768	127.931	*Hynobius leechii*	39.0896	127.449997

Rana amurensis	40.1768	127.931	*Hynobius leechii*	39.0644	127.632004
Rana amurensis	40.1768	127.931	*Hynobius leechii*	39.079601	127.134003
Rana amurensis	40.1768	127.931	*Hynobius leechii*	39.1077	126.124
Rana amurensis	40.1768	127.931	*Hynobius leechii*	39.0742	126.788002
Rana amurensis	40.1768	127.931	*Hynobius leechii*	39.104099	125.834
Rana amurensis	40.1768	127.931	*Kaloula borealis*	39.003799	125.716003
Rana coreana	40.1768	127.931	*Onychodactylus*	39.089802	127.452004
Rana dybowskii	40.188099	126.433998	*Pelophylax chosenicus*	39.0741	125.513001
Rana dybowskii	40.073502	127.426002	*Pelophylax nigromaculatus*	39.020708	125.717619
Rana uenoi-dybowskii	40.188099	126.433998	*Pelophylax nigromaculatus*	39.0877	127.456001
Rana uenoi-dybowskii	40.073502	127.426002	*Rana dybowskii*	39.0746	127.459
Bombina orientalis	40.0186	126.333	*Rana uenoi*	39.044201	125.732002
Bombina orientalis	40.062801	125.485001	*Rana uenoi-dybowskii*	39.0746	127.459
Bombina orientalis	40.054901	127.421997	*Rana uenoi-dybowskii*	39.044201	125.732002
Bufo gargarizans	40.031601	126.170998	*Bufo gargarizans*	38.985199	125.767998
Glandirana rugosa	40.0467	125.073998	*Bufo gargarizans*	38.9468	126.431999
Glandirana rugosa	39.9953	126.124	*Dryophytes japonicus*	38.905721	125.565476
Glandirana rugosa	40.014801	127.464996	*Dryophytes japonicus*	38.995261	125.540474
Hynobius leechii	40.048901	125.238998	*Dryophytes suweonensis*	39.001301	125.718002
Pelophylax nigromaculatus	40.021198	127.418999	*Hynobius leechii*	38.886902	127.647003
Pelophylax nigromaculatus	40.057201	126.182999	*Pelophylax nigromaculatus*	38.908298	125.467003
Pelophylax nigromaculatus	39.968201	124.333	*Pelophylax nigromaculatus*	39.0023	125.724998
Rana dybowskii	40.044498	124.874	*Rana coreana*	38.921171	125.553358
Rana dybowskii	39.978901	126.153999	*Rana coreana*	38.998402	125.824997
Rana dybowskii	40.0186	126.333	*Dryophytes japonicus*	38.866251	125.401032
Rana uenoi-dybowskii	40.044498	124.874	*Dryophytes japonicus*	38.852217	125.412945
Rana uenoi-dybowskii	39.978901	126.153999	*Dryophytes japonicus*	38.850886	125.309697
Rana uenoi-dybowskii	40.0186	126.333	*Dryophytes japonicus*	38.827432	125.874754
Bombina orientalis	39.8643	126.072998	*Dryophytes japonicus*	38.807193	125.636677
Bufo gargarizans	39.878502	127.360001	*Hynobius leechii*	38.787102	127.641998
Bufo gargarizans	39.930698	124.745003	*Hynobius leechii*	38.776402	127.029999
Bufo gargarizans	39.9095	124.882004	*Kaloula borealis*	38.855999	126.133003
Dryophytes japonicus	39.842701	127.556	*Pelophylax chosenicus*	38.865101	125.278818
Kaloula borealis	39.8549	127.417	*Pelophylax chosenicus*	38.866808	125.274351
Pelophylax chosenicus	39.901	124.452	*Pelophylax chosenicus*	38.863121	125.240562
Pelophylax nigromaculatus	39.8717	127.542999	*Pelophylax chosenicus*	38.8367	125.221001
Pelophylax nigromaculatus	39.8106	125.932999	*Pelophylax nigromaculatus*	38.850873	125.309738
Pelophylax nigromaculatus	39.882099	124.264999	*Pelophylax nigromaculatus*	38.864153	125.240826
Pelophylax nigromaculatus	39.842098	124.255997	*Pelophylax nigromaculatus*	38.797401	125.276001
Strauchbufo raddei	39.830399	124.478996	*Bombina orientalis*	38.6567	128.104996
Strauchbufo raddei	39.853901	124.468002	*Bombina orientalis*	38.6222	128.115997
Rana amurensis	39.8312	127.615997	*Bombina orientalis*	38.612	126.035004

Rana amurensis	39.8312	127.615997	*Bombina orientalis*	38.616901	126.581001
Rana amurensis	39.8312	127.615997	*Bufo gargarizans*	38.6441	125.424004
Rana coreana	39.858898	127.538002	*Bufo gargarizans*	38.613098	125.942001
Rana coreana	39.831799	127.611999	*Bufo stejnegeri*	38.633301	128.104996
Dryophytes japonicus	39.782865	125.069206	*Dryophytes japonicus*	38.660099	127.988998
Glandirana rugosa	39.7248	125.869003	*Dryophytes japonicus*	38.685101	128.304993
Hynobius leechii	39.7738	124.907997	*Dryophytes japonicus*	38.653801	126.577004
Pelophylax chosenicus	39.743	124.861	*Dryophytes japonicus*	38.6642	126.042
Pelophylax chosenicus	39.7094	124.922996	*Glandirana emeljanovi*	38.659801	127.989998
Pelophylax nigromaculatus	39.689899	127.345001	*Glandirana emeljanovi*	38.685101	128.304993
Strauchbufo raddei	39.694199	125.820999	*Glandirana rugosa*	38.652199	126.608002
Strauchbufo raddei	39.686401	124.969002	*Glandirana rugosa*	38.619999	126.168999
Bombina orientalis	39.5448	125.475998	*Hynobius leechii*	38.616199	128.108993
Bufo gargarizans	39.607101	127.490997	*Hynobius leechii*	38.645901	128.119995
Bufo gargarizans	39.583599	125.389	*Hynobius leechii*	38.614201	127.369003
Bufo gargarizans	39.540501	125.622002	*Hynobius leechii*	38.723	126.181
Dryophytes japonicus	39.542099	125.431999	*Kaloula borealis*	38.609001	125.401001
Dryophytes japonicus	39.608145	125.558697	*Onychodactylus*	38.636398	127.376999
Dryophytes suweonensis	39.539902	125.457001	*Onychodactylus*	38.6287	128.110992
Glandirana rugosa	39.6544	127.483002	*Onychodactylus*	38.638	128.117004
Hynobius leechii	39.567299	125.834999	*Pelophylax nigromaculatus*	38.657101	127.988998
Hynobius leechii	39.669498	125.403999	*Pelophylax nigromaculatus*	38.685101	128.304993
Pelophylax chosenicus	39.546902	125.457001	*Pelophylax nigromaculatus*	38.637001	126.060997
Pelophylax chosenicus	39.5452	125.449997	*Rana uenoi*	38.6926	126.054001
Pelophylax nigromaculatus	39.6297	127.497002	*Rana uenoi*	38.694698	126.637001
Pelophylax nigromaculatus	39.645302	125.297996	*Rana uenoi*	38.627998	128.108993
Rana coreana	39.594501	125.856003	*Rana uenoi*	38.687401	128.197006
Rana dybowskii	39.6273	127.462997	*Rana uenoi*	38.685101	128.304993
Rana uenoi-dybowskii	39.6273	127.462997	*Rana uenoi-dybowskii*	38.6926	126.054001
Dryophytes japonicus	39.48281	125.479278	*Rana uenoi-dybowskii*	38.694698	126.637001
Dryophytes japonicus	39.471472	125.376714	*Rana uenoi-dybowskii*	38.627998	128.108993
Dryophytes japonicus	39.51183	125.609088	*Rana uenoi-dybowskii*	38.687401	128.197006
Dryophytes japonicus	39.493227	125.56594	*Rana uenoi-dybowskii*	38.685101	128.304993
Dryophytes japonicus	39.528801	127.226997	*Bombina orientalis*	38.495701	125.221001
Dryophytes suweonensis	39.471183	125.376548	*Glandirana rugosa*	38.512501	125.751
Dryophytes suweonensis	39.500306	125.402548	*Hynobius leechii*	38.506802	127.833
Dryophytes suweonensis	39.487254	125.387098	*Hynobius leechii*	38.488899	126.872002
Dryophytes suweonensis	39.492161	125.432633	*Hynobius leechii*	38.5541	125.889999
Dryophytes suweonensis	39.485831	125.47828	*Kaloula borealis*	38.562901	126.108002
Dryophytes suweonensis	39.486587	125.525196	*Pelophylax nigromaculatus*	38.568001	127.674004
Dryophytes suweonensis	39.492596	125.566042	*Rana coreana*	38.486599	125.224998
Dryophytes suweonensis	39.532902	125.466003	*Rana uenoi*	38.494598	125.213997

Dryophytes suweonensis	39.450901	125.481003	*Rana uenoi*	38.549999	127.658997
Hynobius leechii	39.417099	126.360001	*Rana uenoi-dybowskii*	38.494598	125.213997
Hynobius leechii	39.449699	126.152	*Rana uenoi-dybowskii*	38.549999	127.658997
Onychodactylus	39.411499	126.027	*Glandirana emeljanovi*	38.452702	126.176002
Pelophylax chosenicus	39.492637	125.432289	*Hynobius leechii*	38.4575	127.225998
Pelophylax chosenicus	39.471472	125.376714	*Hynobius leechii*	38.369999	127.110001
Pelophylax chosenicus	39.493005	125.563685	*Hynobius leechii*	38.343601	126.584999
Pelophylax chosenicus	39.502701	125.513001	*Hynobius leechii*	38.434299	125.271004
Pelophylax chosenicus	39.452202	125.528	*Hynobius leechii*	38.378502	126.093002
Pelophylax nigromaculatus	39.486585	125.52582	*Kaloula borealis*	38.3605	125.794998
Pelophylax nigromaculatus	39.471992	125.377844	*Pelophylax chosenicus*	38.4454	125.614998
Pelophylax nigromaculatus	39.493045	125.564729	*Rana coreana*	38.440203	125.845677
Pelophylax nigromaculatus	39.480527	125.47816	*Rana coreana*	38.409	125.172
Pelophylax nigromaculatus	39.511915	125.608786	*Rana coreana*	38.387	125.107
Pelophylax nigromaculatus	39.534302	125.442001	*Rana coreana*	38.434	125.322
Pelophylax nigromaculatus	39.528801	127.226997	*Rana coreana*	38.4076	125.68
Pelophylax nigromaculatus	39.530602	127.236999	*Bombina orientalis*	38.3214	127.514
Bufo gargarizans	39.2747	125.999	*Bufo gargarizans*	38.241199	126.753998
Hynobius leechii	39.304699	127.167	*Dryophytes japonicus*	38.2528	126.611999
Bufo gargarizans	39.147499	127.445999	*Glandirana emeljanovi*	38.3186	127.514
Bufo gargarizans	39.147499	127.445999	*Glandirana rugosa*	38.242802	126.792
Bufo gargarizans	39.147499	127.445999	*Hynobius leechii*	38.2798	126.906998
Bufo gargarizans	39.147499	127.445999	*Hynobius leechii*	38.226501	126.658997
Bufo gargarizans	39.147499	127.445999	*Hynobius leechii*	38.334202	126.453003
Bufo gargarizans	39.147499	127.445999	*Hynobius leechii*	38.213501	125.575996
Bufo gargarizans	39.147499	127.445999	*Hynobius leechii*	38.310398	125.396004
Bufo gargarizans	39.147499	127.445999	*Kaloula borealis*	38.2262	126.917999
Bufo gargarizans	39.147499	127.445999	*Kaloula borealis*	38.2262	126.917999
Bufo gargarizans	39.147499	127.445999	*Pelophylax nigromaculatus*	38.322601	127.508003
Bufo gargarizans	39.147499	127.445999	*Pelophylax nigromaculatus*	38.239399	126.598
Bufo gargarizans	39.147499	127.445999	*Rana coreana*	38.213006	126.268705
Bufo gargarizans	39.147499	127.445999	*Rana coreana*	38.2048	125.193001
Bufo gargarizans	39.147499	127.445999	*Rana uenoi*	38.2229	125.214996
Bufo gargarizans	39.147499	127.445999	*Rana uenoi-dybowskii*	38.2229	125.214996
Bufo gargarizans	39.147499	127.445999	*Dryophytes japonicus*	38.0807	126.488998
Bufo gargarizans	39.147499	127.445999	*Dryophytes japonicus*	38.196671	126.278151
Bufo gargarizans	39.147499	127.445999	*Hynobius leechii*	38.183701	126.228996
Bufo gargarizans	39.147499	127.445999	*Hynobius leechii*	38.129101	126.019997
Bufo gargarizans	39.147499	127.445999	*Hynobius leechii*	38.077801	125.827004
Bufo gargarizans	39.147499	127.445999	*Hynobius leechii*	38.1367	125.050003
Bufo gargarizans	39.147499	127.445999	*Rana coreana*	38.158201	126.435326
Bufo gargarizans	39.147499	127.445999	*Bombina orientalis*	37.944901	126.606003

Bufo gargarizans	39.147499	127.445999	*Bombina orientalis*	38.025398	126.454002
Bufo gargarizans	39.147499	127.445999	*Bombina orientalis*	38.028301	125.722
Dryophytes japonicus	39.203612	125.594033	*Bombina orientalis*	37.970798	126.554001
Dryophytes japonicus	39.1475	127.445833	*Bombina orientalis*	37.970798	126.554001
Dryophytes japonicus	39.1475	127.445833	*Bufo gargarizans*	38.019699	126.478996
Dryophytes japonicus	39.1475	127.445833	*Bufo stejnegeri*	38.033828	126.115341
Dryophytes japonicus	39.1475	127.445833	*Dryophytes japonicus*	38.00941	126.632972
Dryophytes japonicus	39.1475	127.445833	*Glandirana emeljanovi*	37.942902	126.603996
Dryophytes japonicus	39.1475	127.445833	*Glandirana rugosa*	38.035801	125.313003
Dryophytes japonicus	39.1475	127.445833	*Hynobius leechii*	37.993999	126.600998
Dryophytes japonicus	39.1475	127.445833	*Hynobius leechii*	38.040901	125.473999
Dryophytes japonicus	39.1475	127.445833	*Kaloula borealis*	38.043098	125.653999
Dryophytes japonicus	39.1475	127.445833	*Onychodactylus*	38.055698	125.716003
Dryophytes japonicus	39.1475	127.445833	*Pelophylax chosenicus*	37.9534	126.671997
Dryophytes japonicus	39.1475	127.445833	*Pelophylax nigromaculatus*	37.950901	126.491997
Dryophytes japonicus	39.1475	127.445833	*Rana coreana*	37.966398	126.375816
Dryophytes japonicus	39.1475	127.445833	*Rana coreana*	38.068699	126.480003
Dryophytes japonicus	39.244044	125.808749	*Rana coreana*	37.955101	126.632004
Dryophytes japonicus	39.188361	125.730404	*Bombina orientalis*	37.816799	126.57
Dryophytes japonicus	39.234749	125.903276	*Bufo gargarizans*	37.9342	126.622002
Glandirana rugosa	39.147499	127.445999	*Dryophytes japonicus*	37.932598	126.5
Glandirana rugosa	39.147499	127.445999	*Dryophytes suweonensis*	37.909	126.66
Glandirana rugosa	39.147499	127.445999	*Kaloula borealis*	37.924099	126.529999
Glandirana rugosa	39.147499	127.445999	*Dryophytes japonicus*	42.236401	130.360001
Glandirana rugosa	39.147499	127.445999	*Pelophylax nigromaculatus*	42.236401	130.360001
Glandirana rugosa	39.147499	127.445999	*Pelophylax nigromaculatus*	42.223	130.488007
Glandirana rugosa	39.147499	127.445999	*Pelophylax nigromaculatus*	42.2281	130.556
Hynobius leechii	39.251701	126.831001	*Pelophylax nigromaculatus*	42.303001	130.483993
Hynobius leechii	39.147499	127.445999	*Pelophylax nigromaculatus*	42.2999	130.457993
Onychodactylus	39.147499	127.445999	*Pelophylax nigromaculatus*	42.261501	130.401001
Onychodactylus	39.147499	127.445999	*Pelophylax nigromaculatus*	42.246201	130.468002
Onychodactylus	39.147499	127.445999	*Pelophylax nigromaculatus*	42.273399	130.544998
Onychodactylus	39.147499	127.445999	*Pelophylax nigromaculatus*	42.3223	130.470001
Onychodactylus	39.147499	127.445999	*Pelophylax nigromaculatus*	42.242199	130.403
Onychodactylus	39.147499	127.445999	*Pelophylax nigromaculatus*	42.257999	130.380005
Onychodactylus	39.147499	127.445999	*Pelophylax nigromaculatus*	42.3302	130.404999
Onychodactylus	39.147499	127.445999	*Pelophylax nigromaculatus*	42.204498	130.417007
Onychodactylus	39.147499	127.445999	*Pelophylax nigromaculatus*	42.219601	130.427994
Onychodactylus	39.147499	127.445999	*Pelophylax nigromaculatus*	42.2896	130.578995
Onychodactylus	39.147499	127.445999	*Pelophylax nigromaculatus*	42.265598	130.526993
Onychodactylus	39.147499	127.445999	*Pelophylax nigromaculatus*	42.3167	130.417007
Onychodactylus	39.147499	127.445999	*Pelophylax nigromaculatus*	42.211102	130.559998

Onychodactylus	39.147499	127.445999	*Strauchbufo raddei*	42.2365	130.360001

1.1.3. Supplementary Table S3

Percent contribution of bioclimate variables. Dataset used for all amphibian species found in the DPR Korea. Species name are abbreviated such as: *Bombina orientalis* Bo, *Bufo gargarizans* Bg, *Bufo stejnegeri* Bs, *Dryophytes japonicus* Dj, *Dryophytes suweonensis* Ds, *Glandirana emeljanovi* Ge, *Hynobius leechii* Hl, *Kaloula borealis* Kl, *Onychodactylus* sp. O sp., *Onychodactylus koreanus* Ok, *Pelophylax chosenicus* Pc, *Pelophylax nigromaculatus* Pn, *Strauchbufo raddei* Sr, *Rana amurensis* Ra, *Rana coreana* Rc, *Rana dybowskii* Rd, *Rana huanrenensis* Rh, *Rana uenoi* Ru, *Salamandrella tridactyla* St

Sp.	Bio1	Bio2	Bio3	Bio4	Bio5	Bio6	Bio7	Bio8	Bio9	Bio10	Bio11	Bio12	Bio13	Bio14	Bio15	Bio16	Bio17	Bio18	Bio19
Bo	1.7526	19.9029	2.0648	1.8516	8.0358	1.7483	2.445	2.6646	0.939	12.4349	0.1393	17.7179	9.2413	3.238	10.365	3.0275	1.3429	0.4805	0.6079
Bg	1.0306	12.3662	2.6233	4.7437	36.7349	1.5603	3.8289	5.6767	0.3711	1.6603	0.7356	3.9974	9.4722	2.6515	3.7139	4.7265	1.9366	1.88	0.2903
Bs	2.9821	2.7952	1.18	0.5745	5.9469	2.0642	1.213	25.9101	0.6504	4.038	0.1575	6.3062	8.1608	0.3343	5.6414	30.2855	0.6676	1.0664	0.0259
Dj	1.1574	13.4523	16.7478	7.8944	16.0711	0.4197	0.4177	2.8381	0.3889	3.5911	0.5038	20.934	2.9234	5.7191	2.2202	2.8302	0.9372	0.6128	0.3408
Ds	0.632	7.8953	23.6776	22.5545	25.6349	0.0338	1.6945	0.9566	0.0614	1.5539	0.0001	1.7251	6.9483	0.276	1.3878	2.4533	0.0221	2.153	0.3399
Ge	8.946	8.3482	1.1846	2.9992	8.7736	8.497	1.215	6.9685	0.3575	11.351	0.2972	15.0352	3.7282	9.2983	7.4354	3.7112	0.5399	0.7008	0.613
Hl	1.6282	6.4155	0.8409	2.6241	13.4705	9.5226	2.0772	2.0115	1.9221	8.8864	0.3148	5.1248	25.6126	9.1193	3.5824	1.6972	3.0912	2.0093	0.0495
Kb	3.2721	5.8536	13.929	2.0218	7.4075	1.0594	3.188	7.5763	4.0471	16.817	1.1961	12.7096	9.1109	0.4836	4.6713	0.647	1.7029	1.0556	3.251
O sp.	3.32	4.9919	5.127	3.387	4.8923	0.3229	0.3008	7.8304	0.2829	21.9487	2.9819	4.2873	3.7051	18.5929	0.6294	9.2007	0.207	7.4323	0.5593
Ok	0	7.3609	5.3783	0.5283	4.6322	0.7651	0.5841	4.3468	0.7331	11.2881	1.919	10.8218	17.6297	25.3695	0.1026	3.7919	0.6695	0.4508	3.6282
Pc	4.5261	19.8148	3.0277	24.1502	7.947	0.0445	0.5723	4.4519	0.1328	14.0539	0.1029	8.2611	8.7441	0.8341	0.5273	1.2815	0.4326	0.0675	1.0276
Pn	0.2726	13.501	9.4034	10.8918	6.6315	6.041	2.7174	1.7658	0.6686	1.8424	0.2252	6.8792	9.039	18.4781	3.7859	1.7289	4.5288	0.7529	0.8464
Sr	0.0298	2.192	31.6799	0.4753	0	0	0	0	0	2.1819	0.0675	0.5334	0	0.9083	37.3047	0.3613	23.7226	0.0258	0.5174
Ra	3.3548	4.9455	0.7302	2.0383	0.7873	0.5758	0.4822	1.1333	25.4582	28.1177	0.9479	0.9744	1.9002	2.4756	4.9274	1.9973	0.173	12.4402	6.541
Rc	0.0449	27.0921	4.1694	0.9465	2.186	0.2267	3.6346	32.1756	0.5657	2.4824	0.4579	4.3802	5.1538	2.425	1.1697	5.2782	3.2093	2.3322	2.0699
Rd	1.2097	1.0562	0.0963	0.4489	3.2118	2.4132	0.4981	1.765	0.19	3.4206	1.669	16.0668	2.7043	3.0119	1.1421	4.1216	33.6126	2.318	21.0441
Rh	1.5791	5.3738	0.0388	0.3861	0.4217	0.1008	1.8107	1.5472	17.0882	3.2543	0.2896	0.3852	32.4522	15.2775	3.2527	0.0017	2.1493	14.5188	0.0724
Ru	2.0497	3.2202	1.5823	0.9487	5.6562	8.8322	4.9909	7.5451	0.3633	12.4509	0.1928	4.4719	3.7138	33.5799	4.829	4.7531	0.4453	0.1705	0.2046
St	0.7935	0.1514	0.2259	0	5.202	0.3326	0.1548	0.9715	2.007	59.5349	0.8688	1.5673	24.6413	0.2267	0.0606	2.099	0.0875	1.0747	0.0005

1.1.4. Supplementary Table S4

Permutation importance of bioclimate variables. Dataset used for all amphibian species found in the DPR Korea. Species name are abbreviated such as: *Bombina orientalis* Bo, *Bufo gargarizans* Bg, *Bufo stejnegeri* Bs, *Dryophytes japonicus* Dj, *Dryophytes suweonensis* Ds, *Glandirana emeljanovi* Ge, *Hynobius leechii* Hl, *Kaloula borealis* Kl, *Onychodactylus* sp. O sp., *Onychodactylus koreanus* Ok, *Pelophylax chosenicus* Pc, *Pelophylax nigromaculatus* Pn, *Strauchbufo raddei* Sr, *Rana amurensis* Ra, *Rana coreana* Rc, *Rana dybowskii* Rd, *Rana huanrenensis* Rh, *Rana uenoi* Ru, *Salamandrella tridactyla* St

Species	Bio1	Bio2	Bio3	Bio4	Bio5	Bio6	Bio7	Bio8	Bio9	Bio10	Bio11	Bio12	Bio13	Bio14	Bio15	Bio16	Bio17	Bio18	Bio19
Bo	2.85	14.7158	4.5579	3.0885	3.1353	11.9531	13.2741	1.1917	1.6448	9.4556	1.1973	6.5718	2.8653	8.7523	4.5347	7.5461	1.3629	0.3352	0.9677
Bg	1.2477	3.1467	1.1513	1.7336	19.0202	9.6164	0.6872	5.2053	2.2573	2.4401	2.0901	4.362	9.5699	15.3862	2.9483	12.4667	3.6551	2.7693	0.2464
Bs	0.2986	7.0237	5.8612	0.4884	8.8917	0.4864	2.248	13.3704	8.9093	1.3471	2.4774	2.3402	11.879	4.063	5.6962	22.5699	0.2895	1.7598	0
Dj	0.902	12.605	2.8145	1.9891	36.9267	1.8023	1.1299	0.5214	0.7754	1.9043	1.7916	4.9005	2.5919	20.5802	3.3332	2.2435	1.3682	1.5634	0.2568
Ds	36.1895	5.2499	8.229	6.0868	8.3731	0.1376	8.3663	3.8759	0	6.0095	0.0142	4.2301	6.6314	0.7784	0.1571	5.0153	0.2555	0.3565	0.0439
Ge	2.4796	4.6247	1.026	0.1706	3.2872	25.1378	3.6687	2.7263	0.617	3.1345	0.9439	7.6899	1.6131	20.3085	10.5525	8.4619	1.3661	0.8151	1.3767
Hl	2.8442	10.0295	4.8227	1.7194	6.7252	13.357	0.4717	1.5065	2.0331	6.7468	0.0235	1.476	9.5179	22.4719	5.1943	3.2752	4.7245	1.8881	1.1728
Kb	0.6457	5.6183	4.1049	1.008	15.8436	0.1548	5.1048	3.4451	13.148	26.3626	1.8792	4.5449	3.62	5.4976	0.6463	0	1.8249	3.0755	3.4758
O sp.	0.0133	2.8932	10.6565	1.2252	2.6837	0.8028	1.278	16.5557	1.1994	10.9376	4.4455	3.7031	3.7133	21.6705	0.3851	5.9869	0.8855	8.1295	2.835
Ok	0	4.4102	4.2165	0.795	1.0171	4.4615	0.0255	8.4975	3.8025	2.9344	0.9503	5.1829	19.3566	12.0794	0.0349	2.8934	8.2481	0.3024	20.7918
Pc	1.2225	23.6313	5.1717	17.3211	1.2606	1.0354	1.4146	28.3271	0.0052	1.7471	0.0009	0.4526	6.3404	7.2635	1.407	1.7174	0.904	0.7776	0
Pn	0.5025	4.7	3.7815	4.9088	1.7275	28.8389	9.4798	0.7349	0.6613	2.2686	0.4425	1.5768	9.1416	11.0475	5.6498	7.4907	4.0732	2.6035	0.3706
Sr	0.0013	7.6887	0.0211	3.5215	0	0	0	0	0	15.2122	0	0	0	0	2.6222	2.2668	68.6662	0	0
Ra	0.6161	1.9403	2.1359	3.7905	3.6251	1.0468	0.2052	1.748	10.408	25.3432	1.1316	3.1601	5.9633	3.2036	4.7688	4.6783	0.272	12.6493	13.314
Rc	0.1857	3.2616	0.1546	0.4712	14.3479	0.5633	2.8371	22.1106	16.2803	0.0637	3.097	6.2455	5.4601	9.009	2.5059	3.4233	0.1104	9.8447	0.0282
Rd	2.7784	1.5536	0.7397	5.3571	1.2727	0.6728	0.7515	0.309	0.009	8.767	1.626	0.1565	10.4532	2.5309	0.9742	8.5982	45.1827	2.3729	5.8947
Rh	20.0873	1.4752	0.1117	0.6036	1.4354	0	0	20.6259	23.6666	2.9077	0.2935	1.8147	4.9718	3.2958	0.3821	0	7.5981	10.6257	0.105
Ru	2.4947	3.4869	2.0072	3.6388	3.9589	12.3051	8.5728	4.4205	0.4264	10.1242	0.0504	1.5221	9.5817	25.3042	3.4849	2.5747	3.651	1.3447	1.0505
St	12.4742	0.2441	0.1548	0	26.8806	0	1.2667	0	0.1966	10.2277	4.7205	0	35.6494	5.069	2.8854	0	0	0	0.231

1.1.5. Supplementary Table S5

Training gain with individual bioclimate variables. Dataset used for all amphibian species found in the DPR Korea. Species name are abbreviated such as: *Bombina orientalis* Bo, *Bufo gargarizans* Bg, *Bufo stejnegeri* Bs, *Dryophytes japonicus* Dj, *Dryophytes suweonensis* Ds, *Glandirana emeljanovi* Ge, *Hynobius leechii* Hl, *Kaloula borealis* Kl, *Onychodactylus* sp. O sp., *Onychodactylus koreanus* Ok, *Pelophylax chosenicus* Pc, *Pelophylax nigromaculatus* Pn, *Strauchbufo raddei* Sr, *Rana amurensis* Ra, *Rana coreana* Rc, *Rana dybowskii* Rd, *Rana huanrenensis* Rh, *Rana uenoi* Ru, *Salamandrella tridactyla* St

Species	Bio1	Bio2	Bio3	Bio4	Bio5	Bio6	Bio7	Bio8	Bio9	Bio10	Bio11	Bio12	Bio13	Bio14	Bio15	Bio16	Bio17	Bio18	Bio19
Bo	0.1375	0.1463	0.0754	0.1289	0.1397	0.1069	0.1385	0.1548	0.1242	0.1563	0.1147	0.1838	0.1406	0.0419	0.156	0.147	0.0578	0.1169	0.0551
Bg	0.1573	0.1047	0.0148	0.0769	0.2148	0.1375	0.0891	0.2019	0.1299	0.1961	0.1356	0.0791	0.1069	0.0407	0.053	0.1168	0.0661	0.0898	0.0556
Bs	0.5341	0.148	0.245	0.215	0.4453	0.5442	0.1707	0.5228	0.5333	0.4554	0.5818	0.3752	0.5712	0.0893	0.1597	0.6854	0.0805	0.5671	0.0793
Dj	0.1132	0.1091	0.1337	0.0684	0.126	0.0599	0.0402	0.1351	0.0916	0.1337	0.0722	0.1612	0.0316	0.0699	0.0443	0.0983	0.082	0.0617	0.0759
Ds	0.4739	0.3483	0.4179	0.456	0.5462	0.3104	0.3541	0.4797	0.3417	0.55	0.3622	0.479	0.3144	0.0924	0.2648	0.3798	0.1947	0.3675	0.193
Ge	0.2447	0.0889	0.0242	0.156	0.1657	0.2167	0.1437	0.2081	0.2183	0.2209	0.2109	0.1856	0.0681	0.0841	0.0842	0.0868	0.0719	0.0538	0.0677
Hl	0.1978	0.0599	0.0079	0.1624	0.1859	0.1908	0.1456	0.1843	0.1943	0.2043	0.1877	0.1366	0.1501	0.1071	0.0741	0.1097	0.1132	0.1186	0.1087
Kb	0.219	0.0977	0.1272	0.0965	0.2254	0.1283	0.0618	0.3198	0.1543	0.2589	0.158	0.1525	0.0823	0.0496	0.0446	0.0906	0.1316	0.0817	0.1341
O sp.	0.1978	0.0931	0.1192	0.1276	0.1868	0.1668	0.1053	0.1755	0.1568	0.2271	0.1779	0.1527	0.1678	0.0991	0.0819	0.1921	0.06	0.1849	0.0595
Ok	0.246	0.2083	0.3101	0.284	0.1751	0.2933	0.23	0.1721	0.2974	0.2407	0.3081	0.4383	0.3808	0.3802	0.1606	0.3774	0.3112	0.3866	0.3127
Pc	0.4701	0.2708	0.1723	0.329	0.3943	0.348	0.2858	0.3872	0.3265	0.4414	0.355	0.3235	0.2669	0.0716	0.1831	0.2551	0.1604	0.1829	0.1472
Pn	0.0807	0.1024	0.0989	0.1245	0.0792	0.0805	0.0899	0.0683	0.0998	0.074	0.1002	0.1127	0.0558	0.1602	0.0625	0.1136	0.1905	0.092	0.1829
Sr	0.1378	0.1015	1.8745	1.1102	0.0024	0.3184	0.5765	0.0066	0.4039	0.0002	0.3906	0.7444	0.0077	1.8996	2.3714	0.0629	2.2396	0.0828	2.2403
Ra	0.4565	0.0635	0.4138	0.2818	0.7166	0.2726	0.2117	0.5794	0.3795	0.7374	0.3297	0.4173	0.4712	0.2125	0.1584	0.4807	0.172	0.4672	0.1535
Rc	0.1829	0.2102	0.109	0.0356	0.2261	0.1603	0.0813	0.2865	0.1355	0.2686	0.1429	0.0699	0.1068	0.0427	0.0201	0.1074	0.0511	0.12	0.0444
Rd	0.9652	0.2297	0.308	0.5675	0.7899	0.8517	0.5546	0.8417	0.8381	0.8491	0.909	1.5192	1.0054	1.6795	0.3838	1.2133	1.7986	1.1776	1.7926
Rh	1.2586	0.8927	0.3926	0.9908	0.5659	1.3524	1.0792	0.724	1.3681	0.8607	1.3768	1.3625	1.8631	1.2243	0.3607	1.5069	1.2399	1.8614	1.3379
Ru	0.1494	0.0708	0.0181	0.1921	0.1522	0.1813	0.1698	0.1656	0.1824	0.1665	0.175	0.1711	0.0722	0.2104	0.0719	0.1149	0.1908	0.0884	0.1826
St	1.9987	0.1471	0.0561	0.4434	2.1913	1.3743	0.341	2.2054	1.5464	2.2521	1.6229	1.2907	1.3599	0.8094	0.0205	1.0761	0.8532	1.0095	0.8394

1.1.6. Supplementary Table S6

List of samples and matching sequences used in this study. All sequences are fragments of the 12S ribosomal small sub-unit, with primers from [1] for *Dryophytes* sp. and priers from [2] for *Rana* sp. Ext. stands for extraction round based on the lysis buffer (see methods) and Ta stands for PCR annealing temperature compared to the one recommended. All sequences presented here have unresolved calls in sequencing, that do not prevent species identification, but would not be adequate for submission to GenBank.

ID	Ext	Ta	Sequence	Species
ZISP.14002	1	Ta+2	TTGTTACTTAACTTATACATGCAAGTCTCAGCACCCCTGTGAGAACGCCCTCAATCTTCTTATTGAAACGAGGAGCTGGTATCAGGCACAATTATTAGCCCAAGACACCTAGCCACGCCACATCCACAAGGAAATTCAGCAGTAATTAACATTGAGTATAAGCGTCAGCTTGACTCAGTTAAAGTAAAGAGAGCCGGCAAATATGGTGCCAGCCGCCGCGGTTACACCACGAGGCTCAAATTGATTACTTTCGGCGTAAAGCGTGATTAAAGTTACCCATTAACTATAGTTGTATTTTAACTAAGCTGTGACACGCTTGTTCTTAAGAAACTCAAAAACGAAAGTTACT	*Dryophytes japonicus*
ZISP.14004	1	Ta+2	TTGTTACTTAACTTATACATGCAAGTCTCAGCACCCCTGTGAGAACGCCCTCAATCTTCTTATTGAAACGAGGAGCTGGTATCAGGCACAATTATTAGCCCAAGACACCTAGCCACGCCACATCCACAAGGAAATTCAGCAGTAATTAACATTGAGTATAAGCGTCAGCTTGACTCAGTTAAAGTAAAGAGAGCCGGCAAATATGGTGCCAGCCGCCGCGGTTACACCACGAGGCTCAAATTGATTACTTTCGGCGTAAAGCGTGATTAAAGTTACCCATTAACTATAGTTGTATTTTAACTAAGCTGTGACACGCTTGTTCTTAAGAAACTCAAAAACGAAAGTTACT	*Dryophytes japonicus*
ZISP.13979	1	Ta+2	CTTCACCAGGTAAAGGAGCTGGTATCAGGCACAGATTCCTTGCCCACAACACCTAGTCTCACCACACCCCCAAGGGTACTCAGCAGTGATTAACTTTGCGCATAAGCGACAGCTTGACTCAGTTAGGGAAAACAGGGCCGGCTAACACGGTGCCAGCCGCCGCGGCTACACCGTGGACCCAAGTTGATAATCACCGGCGTTAAGCGTGATTAAAGTTCTATAAAATTAGGGCCAAATTAACACTTAGTAGTATTATGCTTGTTGTTAAGAAGAACACAAACGAAGGTTGC	*Rana uenoi*
ZISP.13969	1	Ta+4	CTTCACCAGGTAAAGGAGCTGGTATCAGGCACAGATTCCTTGCCCACAACACCTAGTCTCACCACACCCCCAAGGGTACTCAGCAGTGATTAACTTTGCGCATAAGCGACAGCTTGACTCAGTTAGGGAAAACAGGGCCGGCTAACACGGTGCCAGCCGCCGCGGCTACACCGTGGACCCAAGTTGATAATCACCGGCGTTAAGCGTGATTAAAGTTCTATAAAATTAGGGCCAAATTAACACTTAGTAGTATTATGCTTGTTGTTAAGAAGAACACAAACGAAAGTTGC	*Rana uenoi*
ZISP.13971	2	Ta+4	CTTCACCAGGTAAAGGAGCTGGTATCAGGCACAGATTCCTTGCCCACAACACCTAGTCTCACCACACCCCCAAGGGTACTCAGCAGTGATTAACTTTGCGCATAAGCGACAGCTTGACTCAGTTAGGGAAAACAGGGCCGGCTAACACGGTGCCAGCCGCCGCGGCTACACCGTGGACCCAAGTTGATAATCACCGGCGTTAAGCGTGATTAAAGTTCTATAAAATTAGGGCCAAATTAACACTTAGTAGTATTATGCTTGTTGTTAAGAAGAACACAAACGAAAGTTGC	*Rana uenoi*
ZISP.13973	2	Ta+4	CTTCACCAGGTAAAGGAGCTGGTATCAGGCACAGATTCCTTGCCCACAACACCTAGTCTCACCACACCCCCAAGGGTACTCAGCAGTGATTAACTTTGCGCATAAGCGACAGCTTGACTCAGTTAGGGAAAACAGGGCCGGCTAACACGGTGCCAGCCGCCGCGGCTACACCGTGGACCCAAGTTGATAATCACCGGCGTTAAGCGTGATTAAAGTTCTATAAAATTAGGGCCAAATTAACACTTAGTAGTATTATGCTTGTTGTTAAGAAGAACACAAACGAAAGTTGC	*Rana uenoi*

ZISP.139<br>76	1	Ta+2	CTTCACCAGGTAAAGGAGCTGGTATCAGGCACAGATTCCTTGCCCACAACACCTAGTCTCACCACACCCCCAAGGGTACTCAGCAGTGATTAACTTTGCGCATA<br>AGCGACAGCTTGACTCAGTTAGGGAAAACAGGGCCGGCTAACACGGTGCCAGCCGCCGCGGCTACACCGTGGACCCAAGTTGATAATCACCGGCGTTAAGCGT<br>GATTAAAGTTCTATAAAATTAGGGCCAAATTAACACTTAGTAGTATTATGCTTGTTGTTAAGAAGAACACAAACGAAAGTTGC	*Rana uenoi*
ZISP.139<br>78	1	Ta	CTTCACCAGGTAAAGGAGCTGGTATCAGGCACAGATTCCTTGCCCACAACACCTAGTCTCACCACACCCCCAAGGGTACTCAGCAGTGATTAACTTTGCGCATA<br>AGCGACAGCTTGACTCAGTTAGGGAAAACAGGGCCGGCTAACACGGTGCCAGCCGCCGCGGCTACACCGTGGACCCAAGTTGATAATCACCGGCGTTAAGCGT<br>GATTAAAGTTCTATAAAATTAGGGCCAAATTAACACTTAGTAGTATTATGCTTGTTGTTAAGAAGAACACAAACGAAAGTTGC	*Rana uenoi*
ZISP.139<br>72	1	Ta+2	CTTCACCAGGTAAAGGAGCTGGTATCAGGCACAGATTCCTTGCCCACAACACCTAGTCTCACCACACCCCCAAGGGTACTCAGCAGTGATTAACTTTGCGCATA<br>AGCGACAGCTTGACTCAGTTAGGGAAAACAGGGCCGGCTAACACGGTGCCAGCCGCCGCGGCTACACCGTGGACCCAAGTTGATAATCACCGGCGTTAAGCGT<br>GATTAAAGTTCTATAAAATTAGGGCCAAATTAACACTTAGTAGTATTATGCTTGTTGTTAAGAAGAACACAAACGAAAGTTGC	*Rana uenoi*
ZISP.139<br>74	2	Ta+4	CTTCACCAGGTAAAGGAGCTGGTATCAGGCACAGATTCCTTGCCCACAACACCTAGTCTCACCACACCCCCAAGGGTACTCAGCAGTGATTAACTTTGCGCATA<br>AGCGACAGCTTGACTCAGTTAGGGAAAACAGGGCCGGCTAACACGGTGCCAGCCGCCGCGGCTACACCGTGGACCCAAGTTGATAATCACCGGCGTTAAGCGT<br>GATTAAAGTTCTATAAAATTAGGGCCAAATTAACACTTAGTAGTATTATGCTTGTTGTAAGAAGAACACAAACGAAAGTTGC	*Rana uenoi*

1. Hua, X.; Fu, C.; Li, J.; Oca, A.N.M.d.; Wiens, J.J. A revised phylogeny of holarctic treefrogs (genus *Hyla*) based on nuclear and mitochondrial DNA sequences. *Herpetologica* **2009,** *65*, 246-259.
2. Yang, B.-T. *et al.* Diversity and phylogeography of Northeast Asian brown frogs allied to *Rana dybowskii* (Anura, Ranidae). *Molecular phylogenetics and evolution* **2017,** *112*, 148-157.
